# Uncertainty-Aware Framework for CT Radiation Dose Optimization in the Active Surveillance of Small Renal Masses: Clinical and Radiological Considerations

**DOI:** 10.3390/diagnostics16060943

**Published:** 2026-03-23

**Authors:** M. A. Elsabagh, Amira Samy Talaat, Dalia Elwi, Shaimaa M. Hassan, Sameer Alqassimi, Esraa Hassan

**Affiliations:** 1 Department of Machine Learning and Information Retrieval, Faculty of Artificial Intelligence, Kafrelsheikh University, Kafrelsheikh 33516, Egypt; mahmoud_mohsen@fci.kfs.edu.eg; 2 Computers and Systems Department, Electronics Research Institute, Cairo 12622, Egypt; amtalat@yahoo.com; 3 Computer Science Department, Faculty of Computers and Information, Mansoura University, Mansoura 35516, Egypt; dalia_elwi@mans.edu.eg; 4 Department of Histology and Cell Biology, Faculty of Medicine, Menoufia University, Shebin El Koum 32511, Egypt; 5 Department of Histology, General Medicine Practice Program, Batterjee Medical College, Aseer 61961, Saudi Arabia; 6 Department of Internal Medicine, Faculty of Medicine, Jazan University, P.O. Box 114, Jazan 45142, Saudi Arabia

**Keywords:** small renal masses, active surveillance, computed tomography, dose optimization, tumor measurement, diagnostic accuracy, interpretable AI, clinical decision-making

## Abstract

**Background:** Active surveillance of small renal masses is challenged by cumulative radiation exposure from repeated CT imaging, raising long-term health concerns. Low-dose CT protocols offer a strategy to mitigate this risk but are limited by uncertainty regarding measurement accuracy and potential effects on clinical decision-making. **Methods:** We propose an uncertainty-aware analytical framework using a multi-observer dataset of 40 paired CT cases (low-dose vs. standard-dose). The methodology combines statistical agreement assessment (concordance correlation coefficient, intraclass correlation coefficient), multi-algorithm machine learning prediction (linear regression, random forest, gradient boosting, and SVR), and integrated uncertainty quantification to evaluate equivalence across imaging protocols. **Results:** Comparative analysis demonstrates near-perfect concordance between protocols (concordance correlation coefficient = 0.9930). Linear regression achieved the highest predictive performance (R^2^ = 0.9933, MAE = 0.4239 mm, MAPE = 2.07%), outperforming more complex ensemble models, highlighting that interpretable models can achieve superior accuracy without compromising reliability. **Conclusions:** Clinically, the framework supports the safe adoption of low-dose CT for longitudinal tumor assessment, preserving measurement fidelity and diagnostic confidence essential for timely intervention or continued surveillance. Radiologically, it ensures robust lesion characterization across protocols while minimizing cumulative radiation exposure, particularly in younger patients. By integrating uncertainty quantification, this approach enhances transparency, informs clinical decision-making, and facilitates personalized, evidence-based surveillance strategies, promoting safer, dose-optimized imaging in the management of small renal masses.

## 1. Introduction

Active surveillance (AS) is an established management strategy for small renal masses (SRMs), with computed tomography (CT) serving as the primary modality for longitudinal assessment due to its high resolution [1,2,3]. However, the diagnostic utility of CT must be balanced against the risks of cumulative radiation exposure and potential radiation-induced malignancies [4,5,6,7]. While low-dose CT (LDCT) protocols utilizing AI-based denoising aim to satisfy the ALARA principle, maintaining measurement fidelity remains critical; inaccuracies in tumor size assessment directly impact clinical intervention thresholds [8,9]. Reproducible and correct tumor measurement is one of the keystones of AS because the under- or overestimation of tumor size may result in either improper delays in intervention or unwarranted intervention [4,5]. Urology and radiology clinical guidelines highlight the importance of justifying and optimizing imaging use, recommending individual imaging intervals and dose optimization plans [2,3]. Clinically, there has been a significant dilemma of the safety of radiation and the accuracy of diagnostic tests that cannot be compromised in the long-term AS programs.

Even though low-dose CT protocols, which are often backed by iterative reconstruction or deep learning-based denoising, have shown good agreement with routine-dose CT for keeping an eye on small renal masses, the evidence base is still not very strong. Recent studies on active surveillance of renal masses have mainly focused on the reproducibility of measurements and agreement between protocols. These studies have shown that significant dose reduction can maintain tumor diameter assessment in controlled settings and with multiple observers [10]. However, agreement statistics by themselves do not yield a decision-support tool that can be used; they do not show how to map low-dose measurements to routine-dose equivalents at the patient level; and they do not reveal how much confidence is needed to interpret small changes over time that could lead to intervention thresholds. This limitation is more important in real life, where surveillance data are not all the same (different scanner models, acquisition parameters, reconstruction/denoising strategies, and reader variability), and where clinical decisions depend on whether size changes are bigger than expected measurement noise. In the same way, previous research on low-dose CT enhancement has mostly been about improving image quality and the accuracy of measurements through reconstruction or denoising. However, this does not automatically mean that quantitative measurements are equivalent across protocols, clear and reliable for long-term monitoring [11]. Simultaneously, uncertainty quantification has advanced in medical imaging and machine learning; however, it is often regarded as a separate subject and is not consistently incorporated into dose-optimization workflows that also encompass agreement evidence and predictive translation across protocols [12]. Thus, we need a single framework that (i) uses agreement and reliability statistics to show that measurements can be swapped, (ii) learns a clear predictive translation from low-dose to routine-dose measurements to help protocols work together, and (iii) gives calibrated uncertainty estimates so that doctors can tell if differences are likely to be clinically significant or just normal variability. This combined approach directly supports confidence-aware decision-making in active surveillance, where the safety benefits of dose reduction must be balanced against the risk of misclassifying growth trajectories. Even though there is more and more evidence that low-dose CT can be used for active surveillance of small renal masses, there is still a big clinical and methodological gap: there is no single, uncertainty-aware framework that can measure inter-protocol agreement, predict measurement equivalence, and give confidence-aware decision support that is suitable for everyday clinical practice. Current studies generally focus on agreement analysis, predictive modeling, or uncertainty estimation separately, which reduces their applicability and clinical relevance. To fill this gap, the current study suggests a unified analytical framework that aims to assess the interchangeability of low-dose and standard-dose CT measurements while clearly accounting for uncertainty and observer variability. The main goals of this work are: (i) to measure agreement, bias, and reliability between low-dose and standard-dose CT tumor measurements; (ii) to create and test predictive models that can accurately estimate standard-dose measurements from low-dose data; and (iii) to include uncertainty quantification to help doctors make decisions based on how sure they are. We hypothesize that low-dose CT measurements exhibit near-perfect concordance with standard-dose measurements, and that a streamlined, interpretable predictive model can attain superior accuracy without sacrificing reliability, thus facilitating safe, dose-optimized imaging strategies for the longitudinal monitoring of small renal masses.

## 2. Materials and Methods

### 2.1. Dataset Description

**Figure 1:**
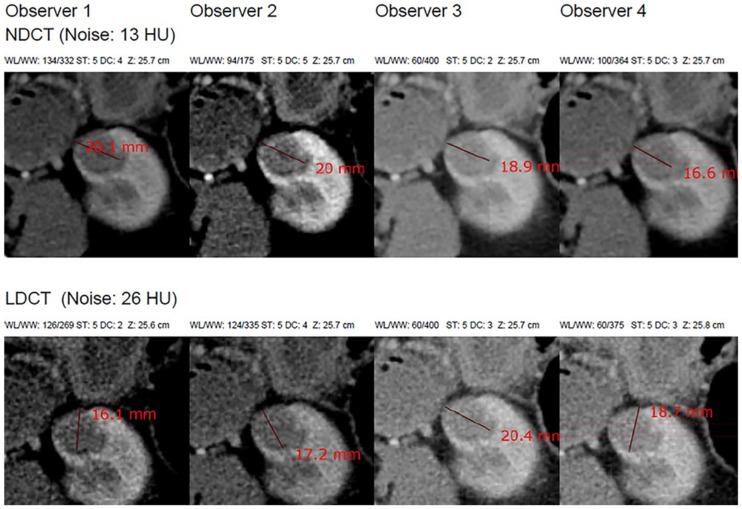
Observer-specific renal tumor diameter measurements from normal-dose CT (NDCT) and low-dose CT (LDCT) for Case 1 show that, despite LDCT having higher image noise, diameter estimates remain consistent across observers and dose levels [13].

The study utilized a publicly available multi-observer dataset designed to evaluate the reproducibility of renal tumor diameter measurements in normal-dose and simulated low-dose computed tomography (CT) that is illustrated in Table 1 and Figure 1. The dataset originates from the 2019 Kidney and Kidney Tumor Segmentation Challenge (KiTS19) and includes contrast-enhanced abdominal CT scans acquired during routine clinical care for patients with renal masses. A final group of 40 patients was selected consecutively by the study investigators from the KiTS19 cohort based on tumor size (≤4 cm for most cases), adequate late-arterial enhancement, and non-infiltrative tumor morphology, with two additional cases (4–5 cm) included to match prior studies. The scans were originally acquired during routine preoperative abdominal CT examinations conducted at Akershus University Hospital, Oslo, Norway. Tumor diameter measurements were performed using a web-based Digital Imaging and Communications in Medicine (DICOM) viewer, with all data collection procedures carried out at the same institution. As this investigation was a retrospective radiological measurement study based on previously acquired clinical imaging data, no additional physical laboratory instruments or chemical agents were used [13].

**Figure 2:**
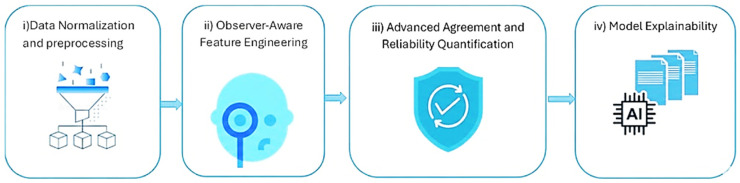
Overview of the proposed framework.

**Figure 3:**
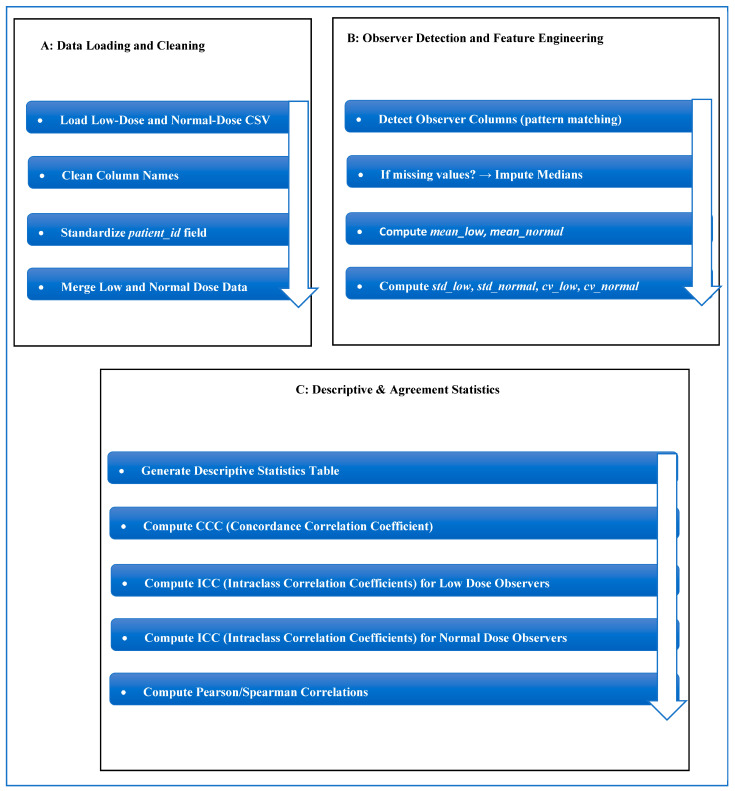
(**A**) The preprocessing pipeline. (**B**) Observer Detection & Feature Engineering workflow. (**C**) Descriptive & Agreement Statistics.

**Figure 4:**
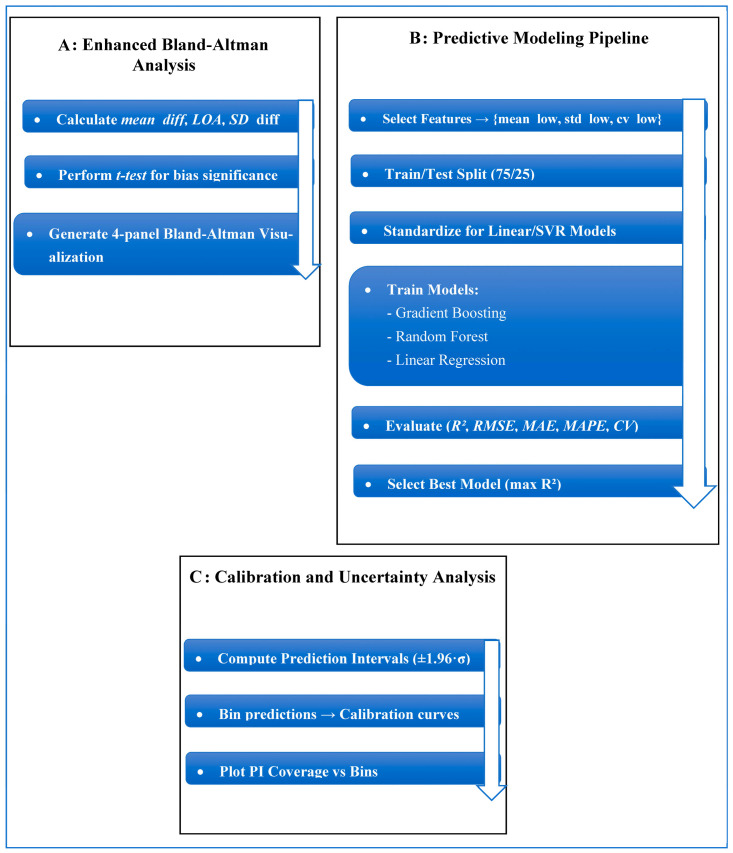
(**A**) Enhanced Bland–Altman Analysis pipeline. (**B**) Predictive modeling workflow. (**C**) Calibration & Uncertainty Analysis.

**Figure 5:**
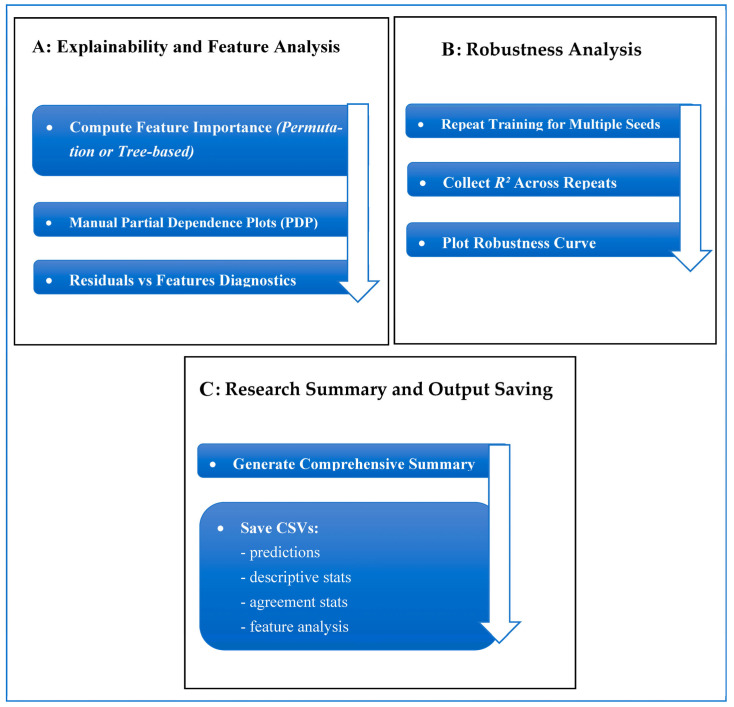
(**A**) Explainability & Feature Analysis. (**B**) Robustness Analysis. (**C**) Research Summary.

The dataset contains two comma-separated value (CSV) files documenting maximum axial tumor diameter measurements performed independently by six radiologist observers. As documented in the original validation study of this dataset [13], the observer group consisted of four board-certified abdominal radiologists with 5 to 10 years of experience after board certification and two radiology residents in their third and fourth years of residency training. Measurements were obtained in both normal-dose CT and simulated 75% dose-reduced CT. The files contain: (1) case number and patient identifier; (2) 6 measurements of observer-specific diameter; and (3) a total of 480 measurements at each dose level of CT. Demographics (age, sex, BMI), tumor histology, tumor size, and CT acquisition parameters (reconstruction method, scan length, tube voltage/current, CTDIvol, DLP, effective dose, and noise levels) are provided as patient-level metadata. The data is freely available on the Mendeley Data Repository [13] and offers a solid basis to measure observer agreement, observer variability, performance of low-dose CT, and quantitative research on reproducibility. The proposed framework, as shown in Figure 2, is a multi-stage analytical and predictive pipeline. The workflow consists of (i) data normalization and preprocessing (Figure 3A), (ii) observer-aware feature engineering (Figure 3B), (iii) advanced agreement and reliability quantification (Figure 3C and Figure 4A), (iv) predictive modeling (Figure 4B), and (v) model explainability and uncertainty quantification (Figure 4C and Figure 5).

### 2.2. Proposed Work Framework

The proposed framework is a multi-stage analytical and predictive pipeline designed to quantitatively evaluate agreement between low-dose and normal-dose radiological measurements and to build a robust predictive model capable of estimating normal-dose values from low-dose observations. The workflow is fully automated and consists of five tightly coupled modules: (i) data normalization and preprocessing; (ii) observer-aware feature engineering; (iii) advanced agreement and reliability quantification; (iv) predictive modeling using multiple machine-learning paradigms; and (v) model explainability, uncertainty quantification, and robustness assessment.

Data normalization includes automated cleaning of metadata, renaming of variables, detection of observer-specific measurement columns, and imputation of missing values. Feature-engineering computes statistical descriptors of low-dose and normal-dose measurements. Agreement evaluation integrates both the concordance correlation coefficient (CCC) and the hierarchical intraclass correlation coefficient (ICC). A full enhanced Bland–Altman module visualizes bias, dispersion, systematic error, and proportional error. The dataset consisted of 40 paired cases, each containing various observer-specific tumor diameter measurements derived from both low-dose and standard-dose CT acquisitions. To make predictions, the data were split into training and testing sets with a fixed 75/25 ratio. This meant that 30 cases were used to train the model and 10 cases were kept for independent testing. A fixed random seed was used during data splitting and model initialization to ensure that the results could be reproduced. We tested the model’s robustness by doing random train–test splits repeatedly, each time keeping the same proportion. We then reported the performance variability across the splits. Observer variability was explicitly addressed through observer-aware feature engineering instead of relying solely on simple averaging. For each case and dose level, individual observer measurements were initially identified automatically, followed by the calculation of descriptive statistics—namely, the mean, standard deviation, and coefficient of variation—across observers. These combined features show both central tendency and how different observers see things differently, and they were used as inputs for the model. We used the full set of observer-specific measurements to do agreement and reliability analyses (CCC, ICC, Bland–Altman) to keep observer-level information. For predictive modeling, we used the aggregated observer-aware features to make case-level predictions that were stable. This method makes sure that observer variability is clearly modeled and included in both statistical and machine learning analyses, which is how clinical measurements are performed in real life.

### 2.3. Machine Learning Algorithms and Architectural Innovation

The choice of machine learning models was based on both methodological rigor and clinical usefulness. Linear regression was intentionally incorporated not solely as a baseline but as a hypothesis-driven model to evaluate whether the correlation between low-dose and standard-dose CT measurements is inherently linear, considering that both seek to estimate the identical physical tumor diameter under diverse noise conditions. In clinical measurement translation tasks, parsimonious linear models are often preferred when they attain similar or greater accuracy, owing to their interpretability, robustness, and ease of implementation. To make sure the comparison was fair and not biased, all models (linear regression, random forest, gradient boosting, and support vector regression) were trained on the same observer-aware feature set, exposed to standardized preprocessing pipeline, tested on identical train-test splits, and given consistent performance metrics (R^2^, MAE, RMSE, and MAPE). We chose hyperparameters based on best practices for each model class, and we used multi-split robustness analysis to double-check the model’s performance. This standardized evaluation framework guarantees that any performance variations observed are indicative of authentic model–data compatibility, rather than inconsistencies in training or evaluation conditions. The gradient boosting regressor is an additive model that constructs a strong predictor by sequentially fitting weak learners (decision trees) to the negative gradients of a differentiable loss function. Given training samples $\left(x_{i},y_{i}\right)$, the model is initialized as:

(1) $$F_{0}(x)=arg\;\underset{\gamma }{min}\;\sum_{i=1}\;L\left(y_{i},\gamma \right)$$

At iteration m, a new tree $h_{m}(x)$ is fitted to the pseudo-residuals:

(2) $$r_{im}=-{\left[\frac{\partial L\left(y_{i},F\left(x_{i}\right)\right)}{\partial F\left(x_{i}\right)}\right]}_{F=F_{m-1}}$$

The model is updated additively:

(3) $$F_{m}\left(x\right)=F_{\left(m-1\right)}\left(x\right)+\nu h_{m}\left(x\right)$$

A random forest constructs an ensemble of B independent regression trees, each trained on a bootstrap sample of the dataset. Prediction for input x is obtained by:

(4) $$\hat{f}(x)=\frac{1}{B}\sum_{b=1}^{B}\;T_{b}(x)$$

This ensemble strategy reduces variance as the expectation of the average predictor satisfies:

(5) $$Var(\hat{f}(x))=\frac{1}{B^{2}}\sum_{b=1}^{B}\;Var\left(T_{b}(x)\right)+\frac{2}{B^{2}}\sum_{b<b^{\prime }}\;Cov\left(T_{b}(x),T_{b^{\prime }}(x)\right),$$

SVR aims to learn a regression function $f(x)=w^{T}\phi (x)+b$. The optimization objective is:

(6) $$\underset{w,b,\xi_{i},\xi_{i}^{*}}{min}\;\frac{1}{2}\|w{\|}^{2}+C\sum_{i=1}^{n}\;\left(\xi_{i}+\xi_{i}^{*}\right),$$

subject to the constraints

(7) $$\begin{array}{cc} y_{i}-\left(w^{⊤}\phi \left(x_{i}\right)+b\right) & \leq \varepsilon +\xi_{i},\\ \left(w^{⊤}\phi \left(x_{i}\right)+b\right)-y_{i} & \leq \varepsilon +\xi_{i}^{*},\\ \xi_{i},\xi_{i}^{*} & \geq 0, \end{array}$$

where the RBF kernel is defined as:

(8) $$K\left(x_{i},x_{j}\right)=exp\left(-\gamma {\left\|x_{i}-x_{j}\right\|}^{2}\right),$$

The linear regression model assumes:

(9) $$\hat{y}=\beta_{0}+\beta_{1}x_{1}+\beta_{2}x_{2}+\beta_{3}x_{3}$$

with parameter estimation via:

(10) $$\beta ={\left(X^{⊤}X\right)}^{-1}X^{⊤}y$$

### 2.4. Architectural Innovations & Equations

The framework automatically extracts all observer measurement columns and computers:

(11) $${Mean}_{\mathrm{low}\;}=\frac{1}{k}\sum_{j=1}^{k}\;o_{ij}^{\mathrm{low}\;}$$

(12) $${\;\mathrm{Mean}\;}_{\mathrm{normal}\;}=\frac{1}{k}\sum_{j=1}^{k}\;o_{ij}^{\mathrm{normal}\;}$$

(13) $${SD}_{\mathrm{low}\;}=\sqrt{\frac{1}{k-1}\sum_{j=1}^{k}\;{\left(o_{ij}^{\mathrm{low}\;}-{Mean}_{\mathrm{low}\;}\right)}^{2}}$$

(14) $$CV_{\mathrm{low}\;}=\frac{{SD}_{\mathrm{low}\;}}{{Mean}_{\mathrm{low}\;}}\times 100$$

CCC quantifies precision and accuracy:

(15) $$\rho_{c}=\frac{2\rho \sigma_{x}\sigma_{y}}{\sigma_{x}^{2}+\sigma_{y}^{2}+{\left(\mu_{x}-\mu_{y}\right)}^{2}}$$

ICC decomposes variance:

(16) $$ICC=\frac{\sigma_{b}^{2}}{\sigma_{b}^{2}+\sigma_{w}^{2}}$$

where variances are estimated using:

(17) $$MS_{\mathrm{between}\;}=\frac{SS_{\mathrm{between}\;}}{n-1}$$

(18) $$MS_{\mathrm{within}\;}=\frac{SS_{\mathrm{within}\;}}{n(k-1)}$$

Bland–Altman mean difference (bias):

(19) $$\bar{d}=\frac{1}{n}\sum \left(x_{i}-y_{i}\right)$$

Limits of Agreement:

(20) $$LOA_{\pm }=\bar{d}\pm 1.96SD_{d}$$

Prediction intervals:

(21) $${\hat{y}}_{i}\pm 1.96\cdot \sigma_{\mathrm{residual}\;}$$

Calibration Error:

(22) $$\mathrm{Calibration}\;{Error}_{b}={\bar{y}}_{\mathrm{actual},b\;}-{\bar{y}}_{\mathrm{pred},b\;}$$

Robustness ($R^{2}$ across S splits):

(23) $$R_{s}^{2}=1-\frac{\sum {\left(y_{i}-{\hat{y}}_{i,s}\right)}^{2}}{\sum {\left(y_{i}-\bar{y}\right)}^{2}}$$

### 2.5. Quantification of Uncertainty and Clinical Interpretation

Uncertainty quantification (UQ) was integrated to explicitly model the anticipated variability linked to predicting routine-dose-equivalent tumor measurements from low-dose CT data. Because the feature space is structured and low-dimensional, and there is a strong linear relationship between low-dose and normal-dose measurements, a frequentist prediction-interval-based approach was used. This strategy allows for clear, distribution-aware uncertainty estimation while keeping clinical interpretability and computational simplicity. Uncertainty was modeled at the level of prediction error instead of image noise, which is more like the uncertainty that comes with estimating tumor size when making decisions about surveillance. We used the estimated residual variance from the training data to make prediction intervals for the best predictive model (linear regression). For every predicted routine-dose-equivalent measurement, a two-sided 95% prediction interval was calculated, encompassing both model uncertainty and intrinsic measurement variability. Interval coverage was empirically validated on the held-out test set by calculating the proportion of observed normal-dose measurements that fell within the predicted intervals. This step of validation made sure that the estimates of uncertainty were accurate and not overly optimistic. The linear regression model completely covered the 95% prediction intervals on the test data. This shows that the uncertainty estimates accurately show how things vary in the real world. In the realm of active surveillance, uncertainty estimates are essential for differentiating genuine tumor growth from anticipated measurement variability. The framework’s UQ outputs are meant to help people understand long-term size changes in a way that considers their level of confidence, not to take the place of clinical judgment. For instance, when a low-dose follow-up scan shows a small increase in tumor diameter, the prediction interval allows doctors to decide if the change is bigger than what they would expect or if it is still within the normal range of measurement variation. Changes that are within the prediction interval can be seen as likely measurement noise. On the other hand, changes that consistently go beyond the uncertainty bounds across serial examinations may call for closer monitoring or intervention.

## 3. Results

Section 3 focuses on the most important findings and clinical interpretation for added clarity and brevity. The tables and figures show the full numerical results.

### 3.1. Descriptive Statistics

The analysis was performed on a dataset comprising 40 paired measurements from low-dose and normal-dose CT imaging protocols. The distribution characteristics were highly similar between the two protocols. The low-dose measurements had a mean of 24.1543 (SD = 8.2095), while the normal-dose measurements had a mean of 24.2487 (SD = 8.0336), indicating nearly identical central tendency and variability. These data are summarized in Table 2.

### 3.2. Inter-Protocol Agreement Analysis

**Figure 6:**
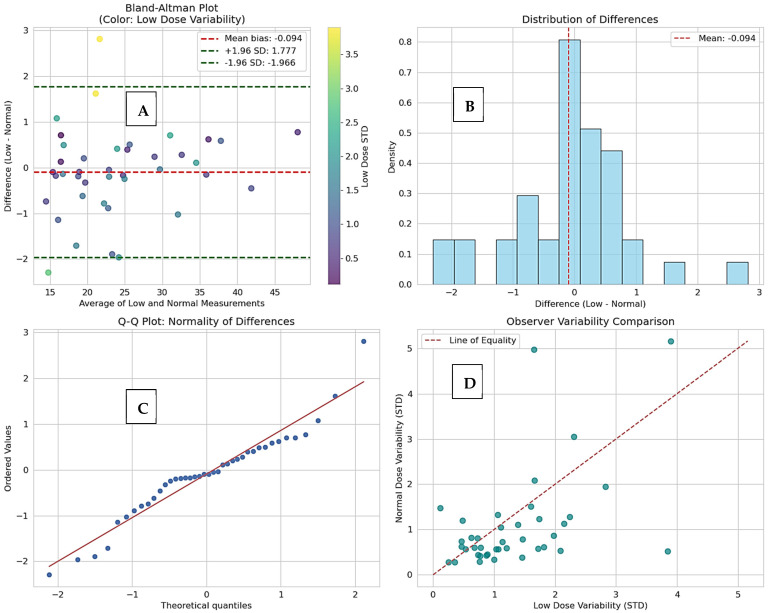
(**A**) The Bland–Altman plot shows minimal bias and close agreement between low-dose and normal-dose measurements. (**B**) The Q–Q plot confirms the normal distribution of the differences. (**C**) The difference distribution further supports normality around the mean bias. (**D**) Observer variability analysis reveals highly similar standard deviations across protocols, demonstrating excellent reliability (ICC > 0.964).

**Figure 7:**
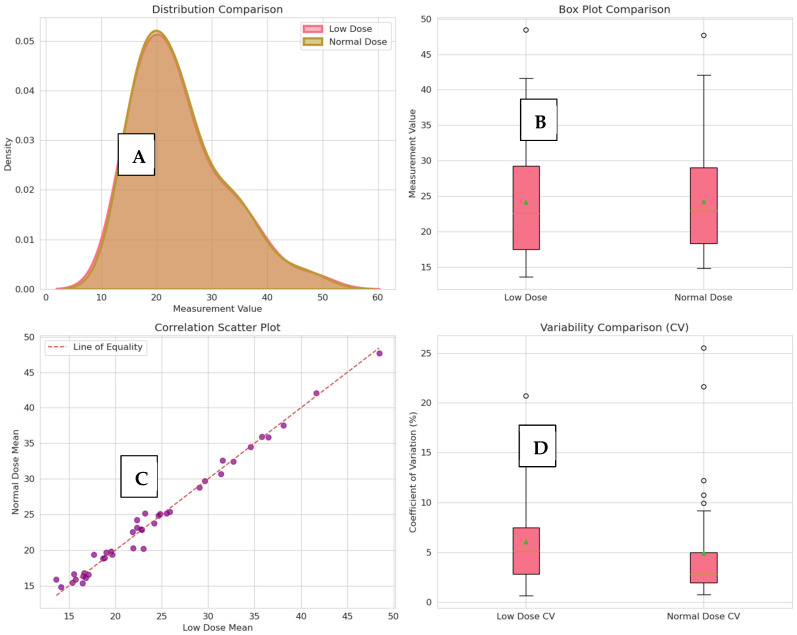
(**A**) Distribution analysis shows nearly identical probability density profiles between low-dose and normal-dose measurements. (**B**) Box plots confirm similar central tendency and variability across both protocols. (**C**) Correlation analysis demonstrates a very strong linear relationship (Pearson r = 0.9933). (**D**) Coefficient variation (CV) results validate consistent precision and reliability throughout the entire measurement range.

Advanced agreement analysis showed almost perfect agreement between low-dose and normal-dose measurements, with very little systematic error and very high reliability across observers. Table 3 shows a summary of the detailed agreement metrics, such as CCC, ICC, correlation coefficients, and error statistics. The observed measurement differences between low-dose and normal-dose CT were significantly lower than clinically relevant growth thresholds utilized in active surveillance protocols, thereby affirming the clinical interchangeability of the two imaging modalities. The measurement reports a concordance correlation coefficient (CCC) of 0.9930. The bias was found to be small and statistically non-significant (−0.0944 mm), with a standard deviation of differences of 0.9547 mm, providing 95% limits of agreement (LoA) between −1.9656 and 1.7768 mm (Figure 6A). High inter-observer reliability (ICC > 0.964) was confirmed for both protocols (Figure 6D). Multi-modal analysis in Figure 7 confirms nearly identical measurement profiles and justifies the clinical interchangeability of low-dose and normal-dose results.

### 3.3. Model Performance

All reported predictive performance metrics are calculated at the patient level, with observer-specific measurements consolidated into aggregated features for each case. During training, no measurements or images from test patients or individual observers were used. Linear regression had the best predictive accuracy and calibration of all the models tested, beating out more complicated nonlinear methods. Table 4 shows a complete quantitative comparison of the performance metrics of the models. As shown in Table 5, Linear Regression achieved an $R^{2}$ of 0.9933 and a Mean Absolute Error (MAE) of 0.4239 mm, with 100% coverage of the 95% Prediction Interval (PI).

### 3.4. Feature Importance and Robustness

**Figure 8:**
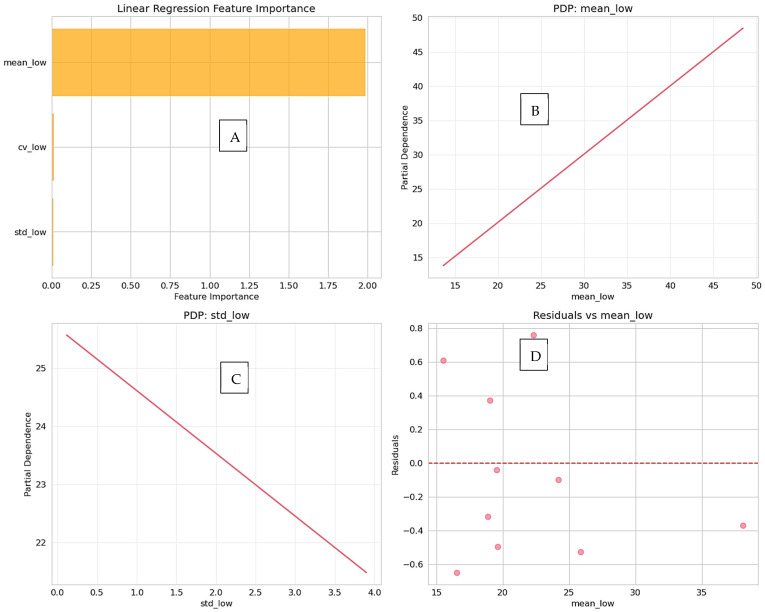
(**A**) Feature importance analysis identifies mean_low as the primary predictor in the model. (**B**) The partial dependence plot (PDP) for mean_low shows a strong linear relationship with predicted normal-dose values. (**C**) The PDP for std_low indicates a minimal marginal contribution to the model output. (**D**) The residuals vs. mean_low plot confirms homoscedasticity and supports appropriate model specification.

**Figure 9:**
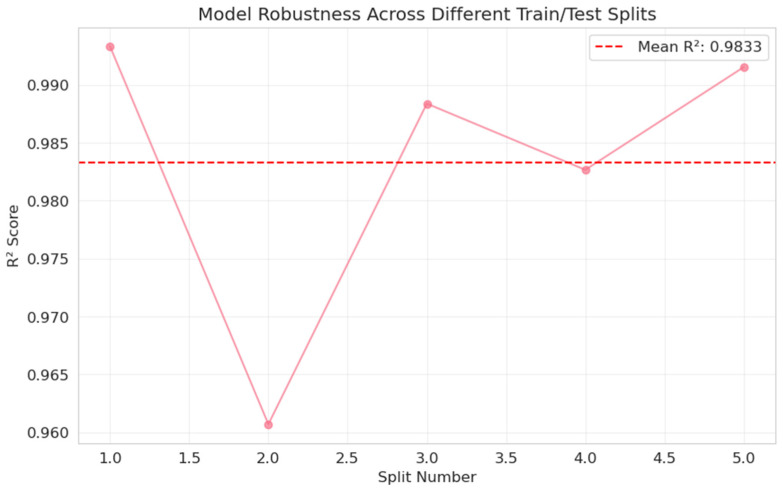
The Linear Regression model’s consistent predictive performance.

A repeated random train–test split analysis was performed to further ensure reliability. This was similar to cross-validation test of robustness. The linear regression model consistently exhibited elevated predictive performance across various splits with distinct test sets, characterized by minimal variability (mean R^2^ = 0.9833 ± 0.0119), signifying that the observed results are not contingent upon a singular data partition. The importance of the features was analyzed in Table 6, where mean_low was found to be the most important predictive feature (score of 1.9868), with a strong correlation to the target (0.9933). The stability of the model is further documented in Table 7 (Robustness across splits) and Table 8 (Summary statistics), showing a mean $R^{2}$ of 0.9833. Interpretability analysis via partial dependence plots (Figure 8) and consistent predictive performance across splits (Figure 9) confirm the model’s reliability.

## 4. Discussion

A precise measurement of the tumor size in the kidneys is essential in diagnosis, designing therapy, and in assessing response to treatment. Standard CT regimes are associated with high-quality images, but they expose patients to significant radiation, which may be very worrisome when used in young children or when they need to undergo repeated scans. The low-dose CT protocols are safer, as they lower the radiation levels but do not compromise the quality of the diagnosis. Our experiment shows that the near-perfect agreement of low-dose CT measurements and standard dose acquisitions can be attained, which serves as an effective route to safer renal tumor scans without affecting the clinical accuracy [14,15,16].

### 4.1. Uncertainty and Clinical Decision-Making

**Figure 10:**
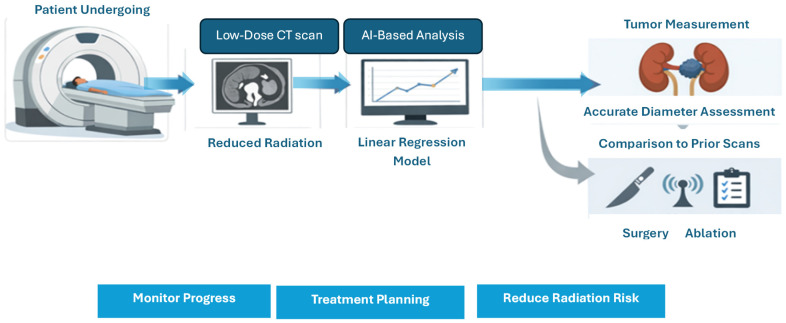
Integration of low-dose CT in routine renal tumor assessment workflow.

Previous research on low-dose CT for monitoring small renal masses has primarily concentrated on establishing measurement interchangeability between reduced-dose and standard-dose acquisitions in a multi-observer context [17,18,19,20]. This includes findings that significant dose reduction can maintain size-based evaluation and that deep learning-based denoising [21,22,23,24,25] enhances low-dose monitoring without compromising clinical interpretability. These studies provide essential clinical reassurance regarding agreement and feasibility; however, they typically stop short of delivering an integrated, deployable framework that (i) quantifies agreement and observer reliability, (ii) learns an explicit predictive mapping from low-dose to routine-dose-equivalent measurements, and (iii) reports calibrated uncertainty to support confidence-aware interpretation of longitudinal changes. In contrast, our contribution is a single, uncertainty-aware pipeline that combines agreement statistics (CCC/ICC/Bland–Altman), multi-model prediction, and uncertainty quantification/robustness testing. This makes it possible to not only check for interchangeability but to translate the results in a way that is clinically meaningful and includes clear confidence estimates. This integration is especially important for active surveillance, where management depends on finding sustained growth that goes beyond the expected range of measurement variability. This is because scanner heterogeneity and multi-reader variation can affect longitudinal consistency. The proposed framework can be used as a post-acquisition decision-support layer in regular active surveillance workflows [26,27]. It works with standard radiological measures and does not change how imaging or reporting is done. After a low-dose CT scan, observer-specific tumor diameter measurements can be entered into the framework. The framework then checks for agreement metrics, makes a predicted routine-dose-equivalent measurement, and gives a prediction interval that shows how uncertain the measurement is. The output helps radiologists and urologists tell the difference between normal changes in measurements and tumor growth that is clinically significant over time [28]. For instance, if a low-dose follow-up scan shows a small increase in size, the framework can tell you if this change is within the expected range of uncertainty or if it is outside of the limits that require closer monitoring or action [9,29]. Figure 10 shows the patient flow beginning with a low-dose CT scan, followed by the AI-based analysis through a Linear Regression model prediction of tumor diameter.

Table 9 indicates the main differences between standard-dose and low-dose CT imaging in some crucial parameters such as radiation exposure, image quality, measurement accuracy, scan time and common clinical applications.

Table 10 depicts the typical tumor size measured in low- and standard-dose CT, revealing that there were minimal differences between the two protocols. The observed differences are minimal, non-systematic, and within the range of clinically acceptable limits, which prove the reliability of low-dose CT in measuring the tumor size accurately and following up [30,31,32].

### 4.2. Clinical and Radiological Considerations

From a clinical standpoint, management decisions during active surveillance are influenced by persistent alterations in tumor size rather than insignificant single-measurement fluctuations. Current urological protocols generally consider growth on the order of several millimeters over time—commonly ≥3–5 mm as potentially actionable. In this context, the mean bias of −0.0944 mm between low-dose and standard-dose CT measurements and the narrow 95% limits of agreement of about ±2 mm are both well below the levels that would be expected to influence clinical decision-making. Consequently, the minor measurement discrepancies noted between dose protocols are improbable to lead to misclassification of tumor growth trajectories or unsuitable alterations in patient management. The discovery that linear regression surpassed more intricate nonlinear models indicates that the low-dose to standard-dose measurement relationship is primarily linear in this context, thereby endorsing the utilization of transparent models for clinically reliable dose-optimized imaging.

### 4.3. Limitations and Future Work

Even though the results are promising, there are some problems with this study that should be noted: (i) The study sample size was relatively small (*n* = 40 cases) due to the lack of publicly available datasets with paired normal-dose and low-dose CT acquisitions for measuring renal tumors by multiple observers. Although this sample size aligns with previous multi-observer imaging studies and adequately demonstrates robust agreement and predictive performance, larger cohorts are required to evaluate scalability, subgroup performance, and infrequent tumor presentations. (ii) The analysis depended on simulated low-dose CT images obtained from routine-dose acquisitions instead of low-dose CT scans that were taken in advance. Simulation-based dose reduction is a widely accepted method for controlled methodological evaluation that enables direct comparison under identical anatomical conditions. However, it may not comprehensively account for all sources of variability present in real-world prospective low-dose imaging, including protocol-dependent noise characteristics and reconstruction discrepancies. To confirm clinical performance, it will be necessary to use true low-dose CT acquisitions for prospective validation.

## 5. Conclusions

This paper establishes a new level for evidence-based dose optimization in medical imaging, providing a rigorous and systematic foundation for safer and more effective imaging practices. The proposed framework is effective in filling the gap between computational analysis and clinical feasibility because it combines the statistical agreement evaluation, predictive modeling, and uncertainty quantification into a single pipeline. Importantly, the low-dose and normal-dose protocols show almost perfect correlation (CCC = 0.9930), and the predictive performance of the study is better using a small and understandable linear regression model (R^2^ = 0.9933, MAE = 0.4239 mm). This observation counters the current belief that complex issues must have complex solutions, which can be of great benefit to clinical readability and implementation. The reliability of the framework is confirmed by the high reliability of low-dose measurements (ICC > 0.964), low levels of bias, and stable robustness of the model when used in different validation splits. These results give great premises to the clinical translation of personalized and low-dose imaging protocols. The fact that linear models can be demonstrated to perform state-of-the-art tasks such as these provides a pathway that is clear and actionable to the reduction of radiation exposure without degrading diagnostic integrity, while importantly preserving the transparency required to make the clinical adoption.

## Figures and Tables

**Table 1:** Case 1: Patient, Tumor, and CT Details.

Parameter	Value
Patient ID (KiTS19)	KiTS-00019
Age (years)	58
Sex	Male
BMI (kg/m^2^)	36.4
Tumor Size (cm)	1.2
Tumor Histology	Oncocytoma
CT System	GE MEDICAL SYSTEMS LightSpeed VCT
Reconstruction Technique (Kernel)	STANDARD
Tube Voltage (kVp)	140
Slice Thickness (mm)	3.75
Scan Range (cm)	31.1

**Table 2:** Descriptive Statistics of Low- and Normal-Dose CT Measurements.

Variable	*N*	Mean (SD)	95% CI	Median [Q1, Q3]	Min–Max	Skewness	Kurtosis
mean_low	40	24.1543 (8.2095)	[21.6102, 26.6985]	22.5774 [17.5154, 29.2217]	13.6433–48.4367	1.0102	0.4978
mean_normal	40	24.2487 (8.0336)	[21.7591, 26.7384]	22.9537 [18.3572, 29.0546]	14.8568–47.6634	1.0116	0.4388

**Table 3:** Advanced Agreement Metrics.

Metric	Value	Interpretation
Concordance Correlation Coefficient (CCC)	0.9930	Almost perfect agreement
ICC: Low-dose observers	0.9642	Excellent reliability
ICC: Normal-dose observers	0.9654	Excellent reliability
Pearson Correlation	0.9933	Very strong relationship
Spearman Correlation	0.9827	Monotonic ranking preserved
Mean Absolute Difference	0.6760 mm	Minimal systematic error
Root Mean Square Difference	0.9474 mm	Very low dispersion

**Table 4:** Model Performance Comparison (Test Set, *N* = 10).

Model	R^2^	MAE (mm)	RMSE (mm)	MAPE	CV R^2^ (Mean ± SD)	95% PI Coverage
Linear Regression	0.9933	0.4239	0.4775	2.07%	0.9632 ± 0.0274	100%
Random Forest	0.9766	0.6657	0.8951	2.87%	0.9034 ± 0.0780	90%
Gradient Boosting	0.9737	0.8149	0.9485	3.72%	0.9146 ± 0.0744	90%
SVR	0.7201	2.2208	3.0943	10.06%	0.4415 ± 0.2253	90%

**Table 5:** Calibration Summary (Best Model: Linear Regression).

Bin	Mean Predicted	Mean Actual	*N*	Calibration Error
1	16.0616	16.6700	1	+0.6084
2	17.0402	16.3910	1	−0.6492
3	19.2817	18.9639	1	−0.3177
4	19.2984	19.6698	1	+0.3713
5	19.9186	19.8773	1	−0.0414
6	19.9293	19.4318	1	−0.4975
7	22.4583	23.2181	1	+0.7598
8	23.8622	23.7645	1	−0.0977
9	25.9144	25.3878	1	−0.5266

**Table 6:** Feature Importance and Statistics.

Feature	Importance Score	Corr with Target	Mean	Std
mean_low	1.9868	0.9933	24.1543	8.2095
cv_low	0.0130	−0.4235	6.1048	4.6161
std_low	0.0099	−0.1557	1.3232	0.8680

**Table 7:** Robustness Across Random Splits.

Split	R^2^ Score
1	0.9933
2	0.9607
3	0.9884
4	0.9827
5	0.9915

**Table 8:** Summary Statistics.

Metric	Value
Mean R^2^	0.9833
Std	0.0119
Min	0.9607
Max	0.9933

**Table 9:** Comparison of Standard-Dose vs. Low-Dose CT for Renal Tumor Imaging.

Feature	Standard-Dose CT	Low-Dose CT	Clinical Implications
Radiation Dose	~10–12 mSv	~1–3 mSv	Low-dose CT significantly reduces patient radiation exposure, which is important for repeated surveillance and younger patients.
Image Quality	High spatial resolution, low noise	Slightly increased noise; sufficient for accurate diameter measurement	Low-dose CT maintains diagnostic quality; AI denoising can further enhance image clarity.
Measurement Accuracy	Reference standard	Near-perfect agreement with standard-dose (CCC ~0.993)	Low-dose CT reliably reproduces tumor size measurements, clinically interchangeable with standard-dose.
Scan Time	Standard	Comparable	No additional workflow burden: low-dose protocols integrate easily into existing practice.
Typical Clinical Use	Initial diagnosis, pre-surgical planning, follow-up	Routine follow-up, active surveillance, dose-sensitive populations	Low-dose CT is preferable for repeated imaging or patients at risk from cumulative radiation exposure.

**Table 10:** Example tumor size differences between low- and standard-dose CT, highlighting the minimal discrepancies observed in practice, consistent with previously reported high agreement and measurement reproducibility between dose protocols.

Tumor ID	Standard-Dose CT (mm)	Low-Dose CT (mm)	Difference (Low − Standard, mm)
1	25.4	25.3	−0.1
2	18.7	18.6	−0.1
3	32.0	32.1	+0.1
4	12.5	12.4	−0.1
5	40.2	40.1	−0.1
6	28.6	28.7	+0.1
7	15.0	14.9	−0.1
8	21.3	21.2	−0.1
9	36.7	36.8	+0.1
10	10.8	10.8	0.0

## Data Availability

The original contributions presented in this study are included in the article. Further inquiries can be directed to the corresponding author.
